# Improving citrus bud grafting efficiency

**DOI:** 10.1038/s41598-023-44832-x

**Published:** 2023-10-18

**Authors:** Randall P. Niedz, Kim D. Bowman

**Affiliations:** https://ror.org/02d2m2044 grid.463419.d 0000 0001 0946 3608 U.S. Department of Agriculture, Agricultural Research Service, U.S. Horticultural Research Laboratory, 2001 South Rock Road, Fort Pierce, FL USA

**Keywords:** Light responses, Plant biotechnology, Plant breeding, Plant physiology

## Abstract

Commercial citrus trees are composed of a scion grafted onto a rootstock. Because grafting is one of the most expensive methods of plant propagation, grafting efficiency is of large practical importance. The purpose of this study was to improve citrus bud-grafting efficiency. The effects of six factors that included BA, Tween-20, DMSO, type of solvent (water or EtOH), cardinal orientation of grafted bud, and type of supplemental light (LED, metal halide, none) on forty-four bud-grafting measures were determined using a multifactor design of experiment approach. Four measures useful for identifying treatments of practical value included the number of rootstock axial buds that formed shoots, the percentage of grafted buds that formed shoots, the length of the longest shoot formed from the grafted buds, and the total leaf area of the grafted bud shoots. The factors that most affected these responses were no supplemental light to minimize the number of shoots from rootstock axial buds, a south orientation and 5 mM BA to maximize the percentage of grafted buds that formed shoots, a north orientation and 5 mM BA to maximize the length of the longest grafted bud shoot, and 5 mM BA to maximize the leaf area of the grafted bud shoots.

## Introduction

Citrus nurseries produce trees by grafting buds from mature cultivar scion trees onto clonally produced rootstocks. Rootstocks are produced using nucellar seedlings, but when seed is limited or the rootstock cultivar is zygotic and produces no apomictic seed, rootstocks are propagated by tissue culture. Grafted trees are then used by citrus growers for planting production groves. Grafted trees are used because of the benefits of a composite rootstock/scion plant. Using citrus rootstocks provide at least three major benefits. One, a shorter juvenility phase compared to seedling-derived trees where juvenility can last for up to 10 years, and sometimes longer. Two, the rootstock confers enhanced resistance to environmental stress and diseases, and thereby allows for production of citrus in areas where the scion on its own roots could not be grown or would grow poorly. Three, enhanced effects on horticultural traits such as tree architecture and fruit yield and quality.

Because grafting is one of the most expensive methods of plant propagation^1^, grafting efficiency is of large practical importance to the citrus industry, particularly bud grafting (budding), the primary method used to graft citrus. There are three components that determine grafting efficiency—healthy budwood that is suitable for grafting, survival of the bud graft, and forcing the bud to form a shoot. Each of these components is affected by many factors, including the skill of the grafter, that determine budding efficiency. Factors that have been studied include the effect of the type of bud graft^2,3^, age of rootstock^3,4^, bud-eye wrapping procedures^5,6^, bud age and position on the bud stick^7^, time of year^7^, and methods to counter the bud suppression effects of apical dominance such as cutting off, topping, lopping, bending, and notching^8,9^ on budding efficiency.

The objective of this study was to improve the bud-grafting efficiency on greenhouse produced rootstock liners. The effects of six factors on measures of bud grafting efficiency were determined. The factors included a cytokinin, a nonionic surfactant, a polar aprotic penetrant, type of solvent, cardinal direction, and type of light arrayed using a multifactor design of experiment (DOE) approach sufficient to identify main, interaction, and curvature effects.

## Results

Twenty-eight responses were measured (Table 1, Supplementary Table S1_Data). Twenty of these responses were comprised of 4 responses measured over time at 2, 4, 6, 8, and 10 weeks. These 4 responses were bud survival, bud growth, number of shoots > 2 mm, and the length of the longest bud shoot. Many of the responses were highly correlated, indicating a lot of redundancy (Supplementary Table S2_Pearsons R). Four responses that were considered useful for identifying treatments that would be practically useful are presented. These included the number of rootstock axial buds that formed shoots (R1), the percentage of buds that formed shoots (R19), the length of the longest shoot formed from a bud (R21), and the total leaf area of the bud shoots (R28). The Pearson r correlation matrix for these four responses is presented (Fig. 1).

**Table 1:** Measured responses, time after budding that each response was measured, and the units of measure.

#	Response	Time after budding	Units
1	Number of rootstock axial buds with sprouts > 2 mm	13 d	Count
2	Bud Survival. 0 = dead, 1 = alive	2 weeks	Count
3	Bud growth. 0 = no growth, 1 = growth	2 weeks	Count
4	Number of shoots > 2 mm	2 weeks	Count
5	Length of longest shoot	2 weeks	mm
6	Bud Survival. 0 = dead, 1 = alive	4 weeks	Count
7	Bud growth. 0 = no growth, 1 = growth	4 weeks	Count
8	Number of shoots > 2 mm	4 weeks	Count
9	Length of longest shoot	4 weeks	mm
10	Bud Survival. 0 = dead, 1 = alive	6 weeks	Count
11	Bud growth. 0 = no growth, 1 = growth	6 weeks	Count
12	Number of shoots > 2 mm	6 weeks	Count
13	Length of longest shoot	6 weeks	mm
14	Bud Survival. 0 = dead, 1 = alive	8 weeks	Count
15	Bud growth. 0 = no growth, 1 = growth	8 weeks	Count
16	Number of shoots > 2 mm	8 weeks	Count
17	Length of longest shoot	8 weeks	mm
18	Bud Survival. 0 = dead, 1 = alive	10 weeks	Count
19	Bud growth. 0 = no growth, 1 = growth	10 weeks	Count
20	Number of shoots > 2 mm	10 weeks	Count
21	Length of longest shoot	10 weeks	mm
22	Length to the highest fully expanded leaf	10 weeks	mm
23	Number of nodes	10 weeks	Count
24	Number of leaves	10 weeks	Count
25	Length to the highest fully expanded leaf. Exclude shoots with < 4 nodes	10 weeks	mm
26	Number of nodes on shoots. Exclude shoots with < 4 nodes	10 weeks	Count
27	Internode length. Shoot length/# of nodes	10 weeks	mm
28	Leaf area	10 weeks	cm^2^

**Figure 1:**
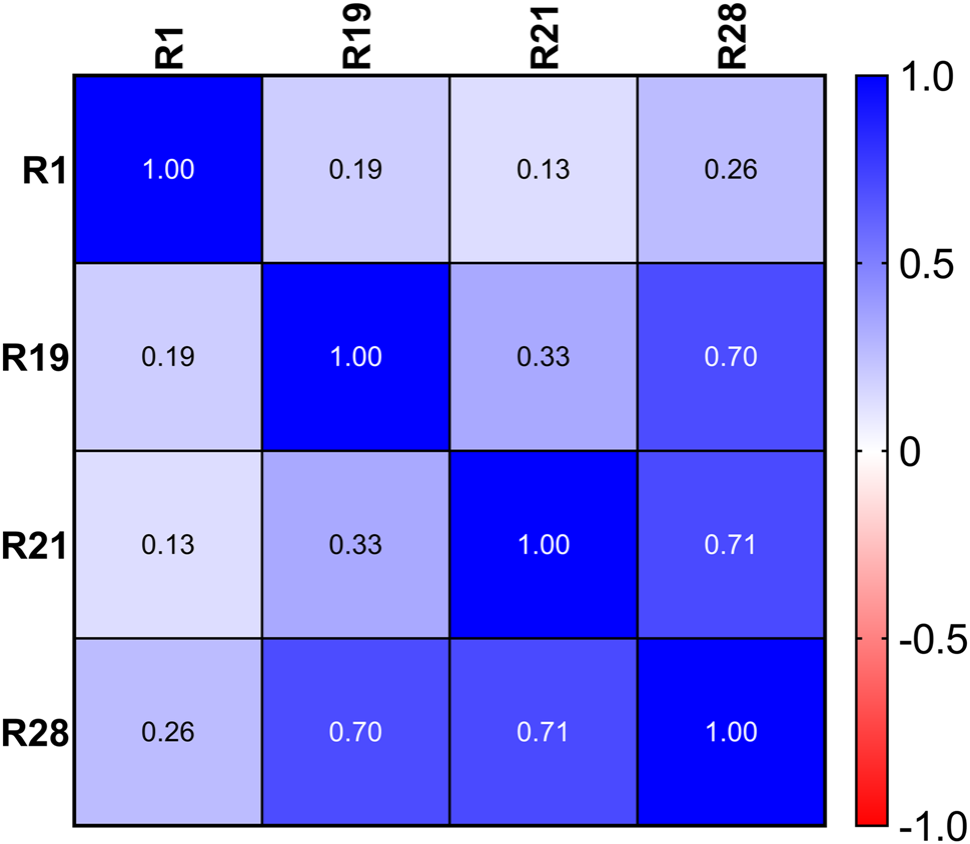
Correlation matrix and heat map of the Pearson r correlations for responses the number of rootstock axial buds that formed shoots (R1), the percentage of buds that formed shoots (R21), the length of the longest shoot formed from a bud (R23), and the total leaf area of the bud shoots (38).

### Response #1, rootstock axial bud growth

**Figure 2:**
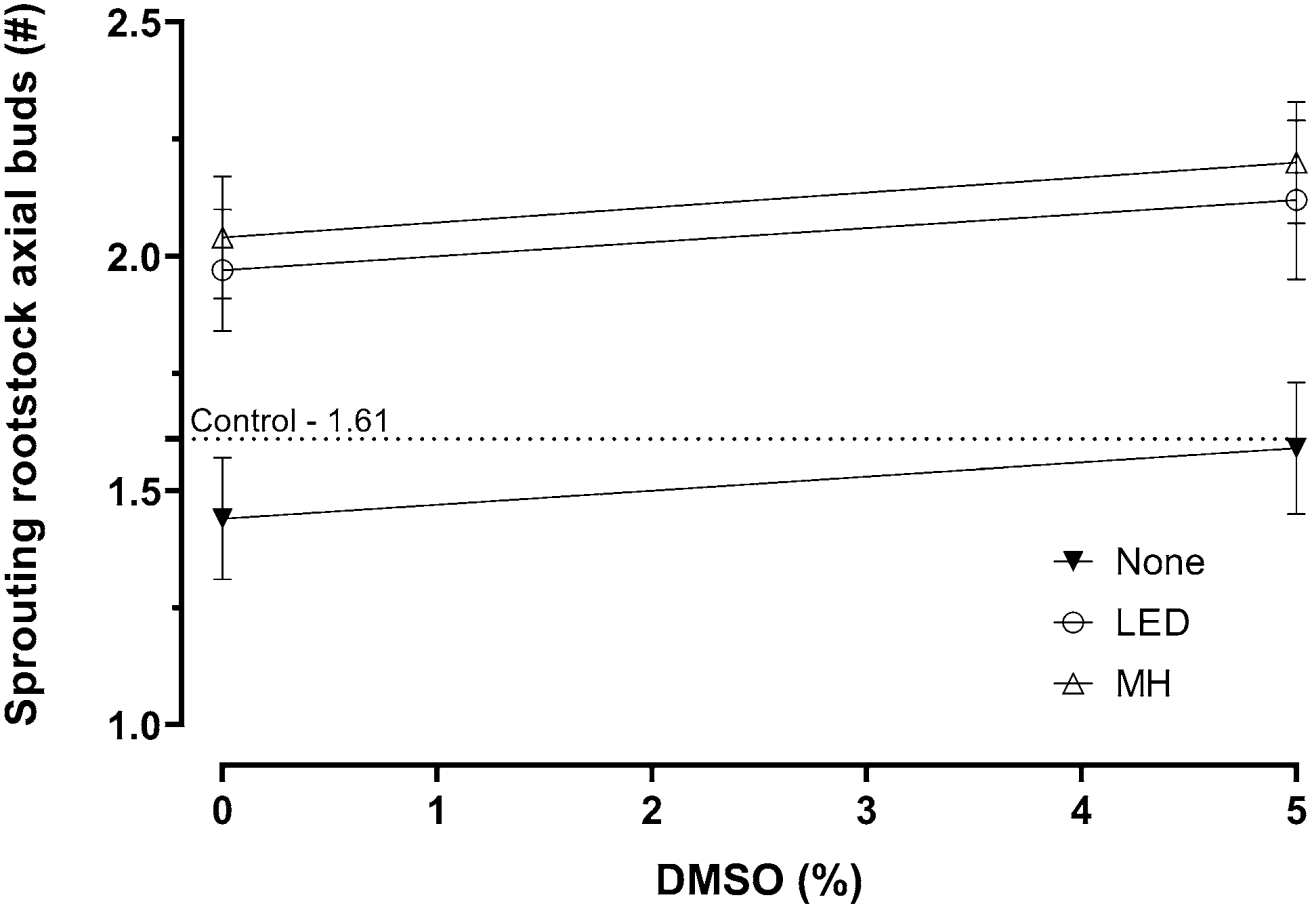
Effect of DMSO and Light on number of axial buds growing from the rootstock 13 days after budding (R1). Rootstock sprouts are not desired and 0% DMSO and no supplemental light minimizes sprouting. Error bars are 95% confidence interval range.

Rootstock axial bud growth is a negative response because it is not desired. The number of axial buds growing from the rootstock 13 days after budding ranged from 1.2 to 2.6 per plant for the treated plants and an average of 1.61 for the two controls (Supplementary Table S1_Data); the wide range suggested that the number of axial buds growing from the rootstock was affected by the factors. The factor settings that resulted in the fewest number of axial buds of 1.2 was design point #32–5 mM BA, 0% Tween-20, 0% DMSO, Water, North, and no supplemental Light. A summary of the ANOVA, lack-of-fit test, three R^2^ statistics, and adequate precision statistic for the number of nodes on grafted shoots is presented (Table 2). The best fitting model was a reduced linear response surface obtained by forward selection using Akaike’s Information Criterion (AICc)^10^. The data satisfied the normality assumption per the Box Cox analysis^11^ and did not require transformation. The lack-of-fit test was not significant (*p* = 0.62) indicating that additional variation in the residuals could not be removed with a better model, the three R^2^ statistics were clustered with a difference less than 0.4 between R^2^ and R^2^_predicted_, and the adequate precision statistic of 13.56 was greater than 4 as recommended^12^. The overall model was highly significant (*p* = 8.18E-09), indicating significant factor effects on the number of axial buds growing from the rootstock. The ANOVA revealed 2 significant terms, DMSO and Light. Light had the single largest effect that included a linear main effect with a *p*-value of 3.47E-09. Light was identified as the primary factor driving the number of axial buds growing from the rootstock. The model predicted that the fewest number of sprouting rootstock axial buds occurs at factor settings of DMSO (0%) and no supplemental Light (Fig. 2). The predicted value is 1.44, a 10% reduction in the number of axial buds formed. BA, Tween-20, Carrier and orientation could be set at any level as they had no significant effects.

**Table 2:** ANOVA model terms, *p*-values (Prob. > *F*), lack-of-fit, and *R*^2^ statistics for the responses # of sprouting rootstock axial buds, percentage of buds with shoots, the length of the longest bud shoot, and total leaf area of bud shoot.

Source	# Axial buds	% Bud growth	Longest shoot length	Total leaf area
	**(R1)**	**(R19)**	**(R21)**	**(R28)**
Model*	8.18E−09	1.63E−08	3.03E−12	1.45E−08
A–BA	–	0.0002	1.72E−13	2.39E−09
B–Tween-20	–	0.5079	–	0.8577
C–DMSO	0.0384	0.1611	–	–
D–Carrier	–	0.0860	–	–
E–Orientation	–	3.47E−09	0.2834	3.51E−05
F–Light	4.59E−09	0.1205	0.0001	0.0025
AE	–	–	0.0003	–
AF	–	–	–	0.0484
BF	–	0.0024	–	0.0248
CD	–	0.0199	–	–
DE	–	0.1188	–	–
A2	–	–	7.33E−05	0.0133
Lack of fit**	0.62	0.99	0.51	0.99
*R*^2^	0.66	0.84	0.85	0.82
*R*^2^ adjusted	0.63	0.78	0.82	0.76
*R*^2^ predicted	0.55	0.67	0.75	0.64
Adequate precision***	12.32	11.35	16.39	12.13
Model type****	Reduced linear	Reduced quadratic	Reduced quadratic	Reduced quadratic
Data transformation	none	none	none	Square root + 16.55

*The F value for the overall model and the probability of obtaining a larger F value. The overall model is a reduced quadratic that included four terms (Table 3).

**A *p* > 0.05 indicates no additional signal was detected that might be accounted for using a better model.

***A signal-to-noise statistic where a value greater than 4 indicates the model is adequate for making predictions.

****Model reduction by forward selection using Akaike’s Information Criterion (AICc) ^10^.

### Response #19, buds that formed shoots

**Figure 3:**
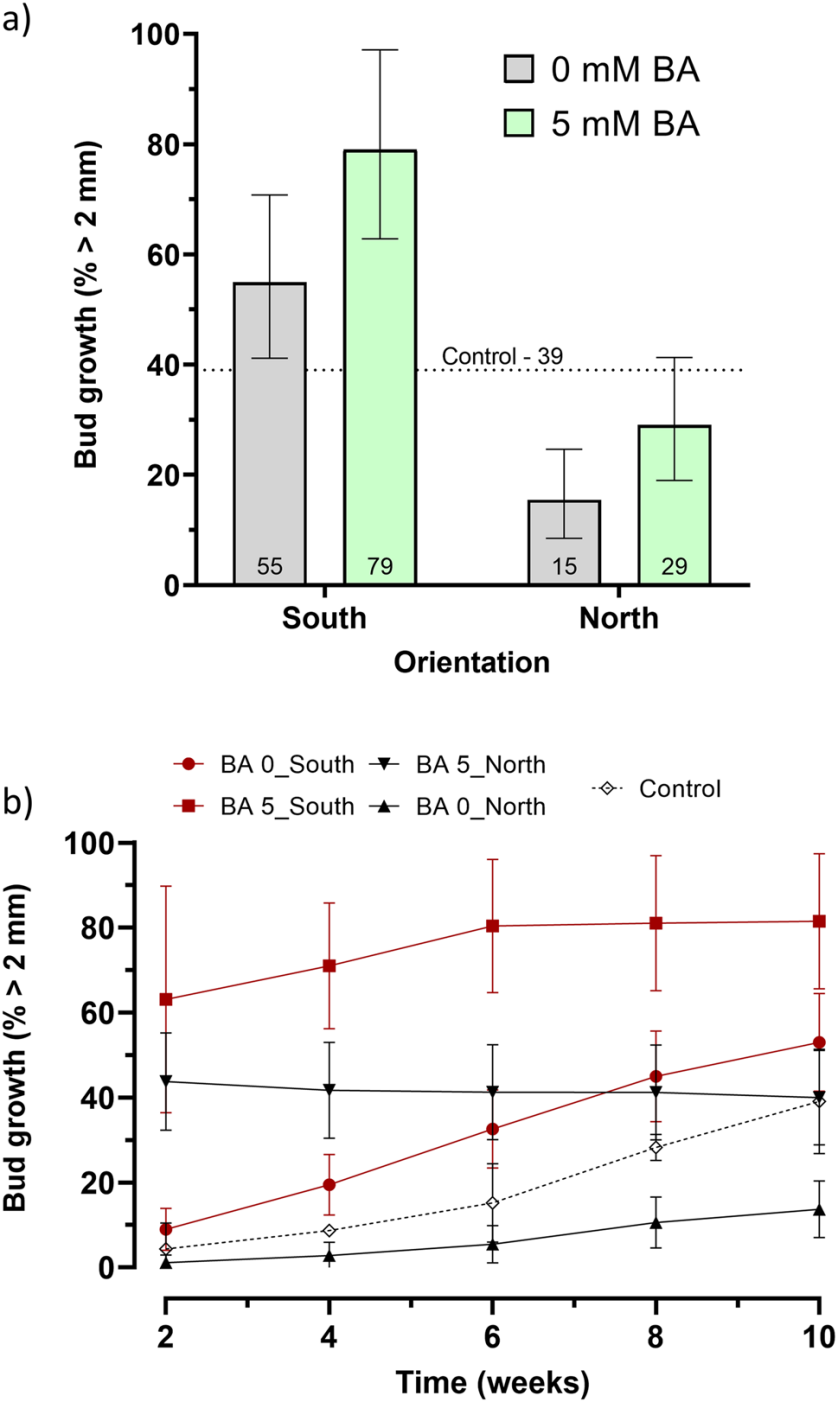
Effect of orientation and BA, the two largest effects, on the proportion of buds that grew (R19). Remaining factors were set at levels to maximize bud growth. Remaining factor settings: Tween-20 (0%), DMSO (0%), Carrier (EtOH), and Light (none). (**a**) Bar graph of Orientation and BA effects. (**b**) Percentage of buds that formed shoots over time at 2, 4, 6, 8, and 10 weeks. Error bars are 95% confidence interval range.

The proportion of buds that form shoots is a positive response. Ideally, 100% of the grafted buds would form shoots. The proportion of grafted buds that were growing after 10 weeks ranged from 0 to 0.77 (Supplementary Table S1_Data) or expressed as percentages 0 to 77% and an average of 39% for the two controls; the wide range suggested that the percentage of grafted buds that grew were affected by the factors. The factor settings that resulted in the greatest percentage of buds of 75% that formed shoots was design point #31–5 mM BA, 0% Tween-20, 0% DMSO, Ethanol, South, and metal halide supplemental Light, a 92% increase over the control of 39%. A summary of the ANOVA, lack-of-fit test, three R^2^ statistics, and adequate precision statistic for 10-week bud growth is presented (Table 2). The best fitting model was a reduced quadratic response surface obtained by forward selection using Akaike’s Information Criterion (AICc)^10^. The data satisfied the normality assumption per a Box Cox analysis^11^ and did not require transformation. The lack-of-fit test was not significant (*p* = 0.99) indicating that additional variation in the residuals could not be removed with a better model, the three R^2^ statistics were clustered with a difference less than 0.4 between R^2^ and R^2^_predicted_, and the adequate precision statistic of 11.35 was greater than 4 as recommended^12^. The overall model was highly significant (*p* = 1.63E-08), indicating significant factor effects on total leaf area. The ANOVA revealed four significant terms, BA, orientation, Tween-20 × Light, and DMSO x Carrier. Orientation had the single largest effect with a *p*-value of 3.47E-09 and was identified as the primary factor driving the proportion of buds growing at 10 weeks (Fig. 3a). BA had the second largest effect, though considerably smaller, on buds growing with a *p*-value of 0.0002. The model predicted that the largest percentage of buds that formed shoots occurred at factor settings of 5 mM BA and South orientation. The predicted percentage is 79%, a 102% increase over the control of 39%. The two significant interaction effects were small and included the Tween-20 × Light effect (*p* = 0.0024) where Tween-20 in combination with LED or MH lighting reduced bud growth, but bud growth was increased with no supplemental light. The DMSO x Carrier effect (*p* = 0.0199) showed that bud growth was greater when EtOH was the carrier at the 0% DMSO level and water was the carrier at the 5% DMSO level. The percentage of buds that formed shoots over time at 2, 4, 6, 8, and 10 weeks is shown (Fig. 3b).

### Response #21, length of the longest shoot from each treatment

**Figure 4:**
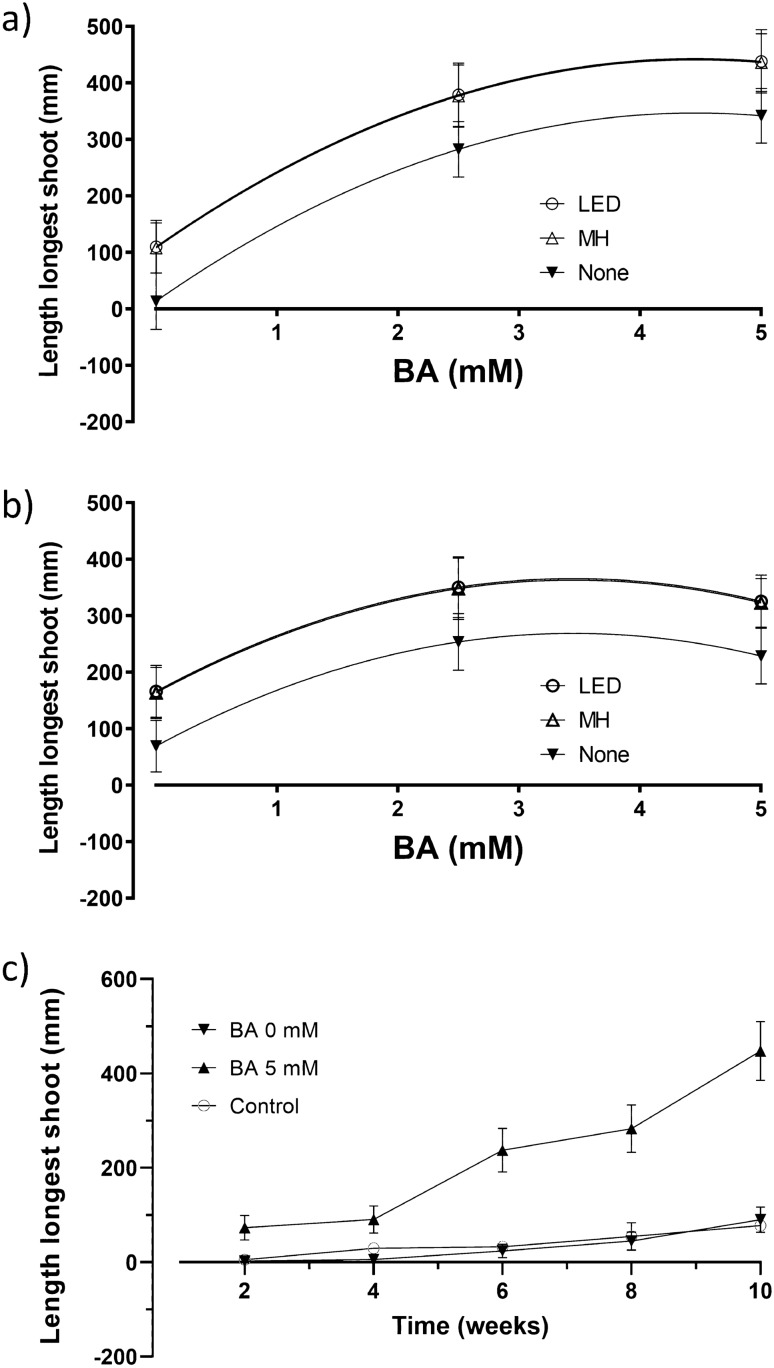
Effect of BA and Light, the two largest effects, on the length of the longest shoot at 10 weeks. The BA x Orientation interaction is shown (**a**) Orientation North. (**b**) Orientation South. Remaining factors were set at levels to maximize bud growth. (**c**) Length of the longest bud shoot over time at 2, 4, 6, 8, and 10 weeks. Error bars are 95% confidence interval range.

The length of the longest shoot is a positive response as it indicates the amount of growth. The longest shoot length from each treatment was measured 10 weeks after bud grafting and ranged from 29 to 494 mm for the treated plants and an average of 77 mm for the two controls (Supplementary Table S1_Data); the wide range in the measures suggested that longest shoot length was affected by the factors. The factor settings that resulted in the longest shoots of 494 mm was design point #7–2.5 mM BA, 2.5% Tween-20, 2.5% DMSO, Aqueous, North, and LED supplemental Light, a 541% increase over the control of 77 mm. A summary of the ANOVA, lack-of-fit test, three R^2^ statistics, and adequate precision statistic for longest shoot length is presented (Table 2). The best fitting model was a reduced quadratic response surface obtained by forward selection using Akaike’s Information Criterion (AICc)^10^. The data satisfied the normality assumption per a Box Cox analysis^11^ and did not require transformation. The lack-of-fit test was not significant (*p* = 0.51) indicating that additional variation in the residuals could not be removed with a better model, the three R^2^ statistics were clustered with a difference less than 0.4 between R^2^ and R^2^_predicted_, and the adequate precision statistic of 16.39 was greater than 4 as recommended^12^. The overall model was highly significant (*p* = 3.03E-12), indicating significant factor effects on 10-week longest shoot length. The ANOVA revealed four significant terms, BA, light, BA x Orientation, and the quadratic effect of BA (BA^2^). BA had the single largest effect that included a positive linear main effect with a *p*-value of 1.72E-13 and a quadratic effect with a *p*-value of 7.33E-05. BA was identified as the primary factor driving the length of the longest shoot 10 weeks after budding. The effect of Light was that longest shoot length was shorter with no supplemental light versus LED or MH lighting (Fig. 4). The model predicted that the longest shoots occurred at factor settings of 4.5 mM BA, North orientation, and LED lighting. The predicted length is 443 mm, a 475% increase over the control of 77 mm. The effect of BA x Orientation was that longest shoot length was greater with 5 mM BA and North Orientation and the shortest with 0 mM BA and south Orientation. The length of the longest bud shoot over time at 2, 4, 6, 8, and 10 weeks is shown (Fig. 4c).

### Response #28, total leaf area of the bud shoot

**Figure 5:**
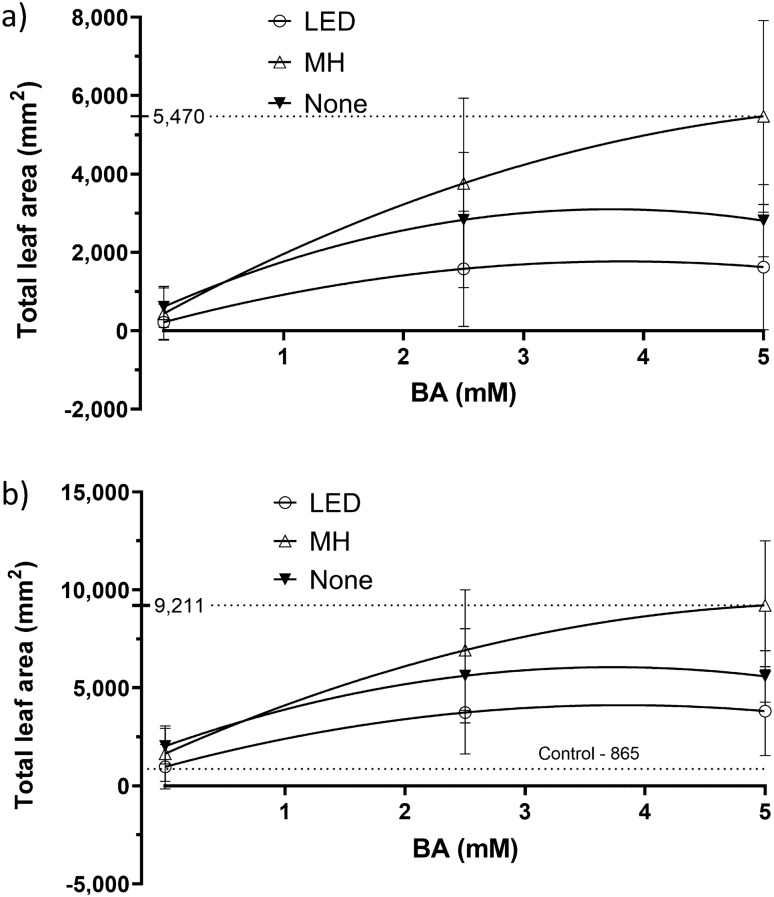
Effect of BA and Light on total leaf area from grafted bud shoots at 10 weeks. The BA x Orientation interaction is shown (**a**) Orientation North. (**b**) Orientation South. Tween-20, the remaining factor in the model, was not significant and was set at 0%. Error bars are 95% confidence interval range.

Total leaf area of the bud shoot is a positive response as it indicates the amount of growth. Total leaf area of the shoot that emerged from each grafted bud was measured 10 weeks after bud grafting and ranged from 0 to 16,548 cm^2^ for the treated plants and an average of 865 cm^2^ for the two controls (Supplementary Table S1_Data); the wide range in the measures suggested that total leaf area was affected by the factors. The factor settings that resulted in the greatest leaf area of 10,932 cm^2^ was replicate design points #8 and #2–5 mM BA, 5% Tween-20, 0% DMSO, Aqueous, Sorth, and metal halide supplemental Light, a 1,163% increase over the control of 865 cm^2^. A summary of the ANOVA, lack-of-fit test, three R^2^ statistics, and adequate precision statistic for total leaf area is presented (Table 2). The best fitting model was a reduced quadratic response surface obtained by forward selection using Akaike’s Information Criterion (AICc)^10^. The data required a square root transformation per the Box Cox analysis^11^. The lack-of-fit test was not significant (*p* = 0.99) indicating that additional variation in the residuals could not be removed with a better model, the three R^2^ statistics were clustered with a difference less than 0.4 between R^2^ and R^2^_predicted_, and the adequate precision statistic of 12.13 was greater than 4 as recommended^12^. The overall model was highly significant (*p* = 1.45E-08), indicating significant factor effects on total leaf area. The ANOVA revealed six significant terms, BA, Orientation, Light, BA x Light, Tween-20 × Light, and the quadratic effect of BA (BA^2^). BA had the single largest effect that included a large linear main effect (*p* = 2.39R-08) and a small quadratic effect (*p* = 0.0133). BA was identified as the primary factor driving total leaf area of the grafted bud shoots, with the linear main effect of BA accounting for most of the variation (Fig. 5). The model predicted that the greatest leaf area of the bud shoots occurred at factor settings of 4.8 mM BA, 5% Tween-20, South orientation, and metal halide Light (Fig. 5). DMSO and Carrier could be set at any level as they had no significant effects. The predicted leaf area is 9,675 cm^2^, a 1,018% increase over the control of 865 cm^2^.

## Discussion

Bud grafting efficiency is economically important to the citrus nursery industry because it is expensive but how commercial citrus scion/rootstock trees are produced. To determine if bud grafting efficiency could be improved, we selected factors that might improve grafting efficiency sufficiently to be of value to commercial citrus tree producers. These factors included components of a solution applied to each bud graft and environmental factors. The experiment was designed to detect and quantify single factor main effects, 2-factor interactions, and the quadratic effects of each factor. The quadratic effects were included to detect curvature. If curvature is present, then the significant quadratic terms will locate the optimal settings for those factors. A response surface design was used as these types of designs are considerably more efficient and provide much more information than running factors singly or using full factorials. Single factor experiments cannot detect interactions and lack the efficiencies that come with internal replication. The full factorial for this experiment would have required 324 treatment combinations and was resource prohibitive; the response surface design used required 38 unique treatment combinations and fit the available greenhouse, plant, and personnel resources.

Of the six factors tested BA had the single largest effect across most of the measured bud grafting responses. BA is a cytokinin and was selected because cytokinins are plant growth regulators that affect cell division and differentiation^13^ and might affect bud grafting.

BA’s effect was positive and including BA in the bud treatment solution both improved the percentage of grafts that grew and resulted in larger shoots. The effect was sufficiently large that commercial citrus nurseries might consider treating grafted buds with BA. When BA was applied to ‘Valencia’ sweet orange (*C. sinensis* (L.) Osbeck) buds grafted onto ‘Troyer’ citrange (*Poncirus trifoliata* (L.) Ref. X *C. sinensis* (L.) Osbeck) budburst was consistently accelerated compared to the control treatment^14^. Though cytokinins are not widely used to enhance bud grafting, they have been shown to stimulate the growth of quiescent buds in various crops^15–21^ including citrus^22^. Some orchids, such as Dendrobium and Phalaenopsis, are prone to forming plantlets from nodes along the flower stem; these plantlets are called keikis and allow for the vegetative propagation of these orchids. Cytokinins stimulate the formation of keikis^23^ and one method of propagation is to use keiki paste^24,25^, a lanolin paste containing a cytokinin, often BA, to enhance the production of keikis.

The primary mechanism by which cytokinins enhances budburst is that they disrupt the auxin-mediated suppression of lateral buds exerted by apical dominance^21,26^. However, the observation that auxin does not enter axillary buds^21,27^ has resulted in an alternative explanation that states that an insufficient supply of sugar suppresses bud growth. The growing shoot tip requires large amounts of sugar, but when the tip is removed by pruning there is a rapid redistribution of sugars to the lateral buds and budburst is observed to occur^28^. Cytokinins may induce bud growth by repressing the expression of BRANCHED1 (BRC1), a key regulatory transcription factor identified in Arabidopsis^29,30^.

Supplemental light strongly promoted the outgrowth of axial rootstock buds. The growth of axial buds from the rootstock is a negative response because the bud shoots must be removed which increases production costs. In general, plants produce fewer lateral shoots when grown under low light or low red to far red proportion (R:FR) compared to plants grown under high light^31,32^. It is also observed when plants are shaded from being grown at high densities^32,33^. This phenomenon is called shade avoidance syndrome^34^. Thus, the stimulation of the axial rootstock buds by the supplemental lighting is not unexpected.

The cardinal orientation of the grafted bud had a strong positive effect on the proportion of buds that grew and the leaf area of the resulting shoots. Cardinal orientation was added as a factor because observations over the years of producing grafted trees for the USDA rootstock breeding program suggested that the cardinal orientation of the grafted buds might be important. One possible explanation for the effect is temperature since the positive effect was observed to occur on grafted buds oriented to the south. Perhaps the temperature gradient between the north and south orientation was sufficient to enhance growth. Temperature is known to affect bud growth and dormancy^35–38^. Regardless of the mechanism of the orientation effect, it is an inexpensive treatment for citrus nurseries to implement.

## Methods

The study was conducted at the U.S. Horticultural Research Laboratory (USHRL) in Fort Pierce, Florida (27.42781350558675, -80.4089433369076). This study used plant material that complied with all relevant institutional, national, and international guidelines and regulations.

### Plant material

The citrus rootstock US-802 (*Citrus maxima* ‘Siamese’ × *P. trifoliata*) was used in this nursery study, because of outstanding field performance and substantial commercial use in Florida^39–42^, and observations of difficulty in obtaining good budbreak and growth with grafted scions on this rootstock in the nursery during winter months. In preparation for the experiment, seeds of US-802 were obtained from certified seed source trees located at the Whitmore Foundation Farm (Groveland, Florida) for the respective rootstocks. These seed were harvested from the source trees in the previous season, treated with 8-hydroxyquinoline sulfate, and stored at 4 °C until use.

### Growing conditions

Seedlings were started in soilless potting mix (Pro Mix BX; Premier Horticulture, Inc., Quakertown, PA), by inserting a single seed in each cell in racks of 3.8 cm × 21 cm cone cells (Cone-tainers; Stuewe and Sons, Tangent, OR). Seedlings were chosen for trueness-to-type and strong growth and were transplanted at two seedlings per 2.54 L pot (Treepots; Stuewe and Sons, Tangent, OR) using the soilless potting mix (Pro Mix BX) at least 8 weeks before start of the experimentation. The plants received a liquid fertilizer application of water-soluble fertilizer (20N-10P-20 K; Peters Professional, The Scotts Company, Marysville, OH, USA) every other week, at a rate of 400 mg N per liter. Between fertilizer applications, plants were irrigated with water as needed. Insecticides and miticides were also applied as needed. Throughout the experiments, the plants were grown in a temperature-controlled greenhouse with mean weekly temperatures of 25–30 °C.

### Budding

On October 19th or November 15th, each of 1120 seedlings were budded with one bud of the sweet orange (*C. sinensis*) scion clone ‘Valencia’ 1-14-19, produced on increase trees in our greenhouses. Grafting of the ‘Valencia’ scion onto the rootstock liner was by inverted T bud, and grafted buds were wrapped with budding tape. Two weeks after budding, the budding tape was removed. On the day the budding tape was removed, bud survival was assessed, and only plants with living Valencia buds were used for the treatments and assessments described below. Six days after the buds were unwrapped, the rootstock was trimmed and looped to force bud growth using methods as previously described^9^. One day after the rootstock was looped and trimmed (21 days from budding), the illumination, orientation, and chemical treatments were applied as detailed below.

### Illumination treatments

Three light treatments were compared: (1) natural sunlight only, (2) natural sunlight plus daylength extended to 16 h using supplemental 1000-W metal halide light (MH; Full Nova, Sunmaster Grow Lamps, Twinsburg, OH, USA) at 155 cm above the inserted buds, and 3) natural sunlight plus daylength extended to 16 h using supplemental 280-W LED light (LED; Optic X3-Pro, Optic Lighting, USA) at 140 cm above the inserted buds. To achieve the 16 h daylength during the time of the study, the supplemental lights were controlled by timer, and switched on from 1:30 am to 8:30 am each day. During the hours when there was no natural daylight and the supplemental lighting was on, average PPFD from the MH light at the height of the bud was 206 µmol m^−2^ s^−1^, and average PPFD from the LED light at the height of the bud was 179 µmol m^−2^ s^−1^.

### Orientation treatments

The potted and budded plant was rotated so that the Valencia bud faced either South or North, and this orientation was continued throughout the experiment.

### Chemical treatments

Treatment solutions were prepared as detailed in Table 4. Solutions were applied with a micro brush (Testor Corporation, Rockford, IL), using one dip in the solution, swipe on inside of tube to prevent drip, and two strokes directly over each bud. The sources of the chemicals used were as follows: 6-Benzylaminopurine (BA)(PhytoTech Labs, Lenexa, KS, USA), TWEEN® 20 (Sigma-Aldrich, St. Louis, MO, USA), Dimethyl sulfoxide (DMSO)(PhytoTech Labs, Lenexa, KS, USA), and 200 proof dehydrated punctilious ethanol (Quantum Chemical Corporation, now Millennium Petrochemicals, La Porte, TX, USA).

### Experimental design

Six factors were varied (Table 3) and twenty-eight responses were measured (Table 1). The experiment was a 6-factor response surface design constructed using 33 points in 3 blocks sufficient for modeling a quadratic polynomial selected by D-optimality criteria (Table 4). Ten additional points were added to augment the design that included 5 lack-of-fit points for detecting cubic curvature and 5 replicate points to provide an estimate pure error. The total number of design points was 43. Two sets of controls were added that were not treated and the grafted buds oriented to the south. The response at each point was estimated from 21 to 28 budwood grafts. The total number of grafted plants, including the two control sets was 1120.

**Table 3:** The six factors used to construct the design space of the experiment.

Factors	Range
(1) BA	0–5 mM
(2) Tween 20	0–5%
(3) DMSO	0–5%
(4) Carrier	Ethanol, Water
(5) Orientation	North, South
(6) Light (supplemental)	None, metal halide, LED

**Table 4:** Six-factor D-optimal quadratic response surface design matrix. The design included 33 model points, 5 lack-of-fit points, and 5 replicated points for pure error estimation.

ID*	Block	Design points (runs)	Factor 1	Factor 2	Factor 3	Factor 4	Factor 5	Factor 6
			BAP	Tween 20	DMSO	Solvent	Orientation	Light type
			mM	%	%	Type	Direction	Type
9	1	1	0	0	5	EtOH	North	MH
11	1	2	5	5	0	Water	South	MH
3	1	3	0	0	0	EtOH	South	None
7	1	4	5	5	0	EtOH	South	LED
7	1	5	5	5	0	EtOH	South	LED
10	1	6	5	5	5	EtOH	South	MH
0	1	7	2.5	2.5	2.5	Water	North	LED
11	1	8	5	5	0	Water	South	MH
1	1	9	0	5	0	EtOH	North	None
8	1	10	0	5	5	Water	South	LED
5	1	11	5	0	5	Water	South	None
2	1	12	2.5	5	5	Water	North	None
4	1	13	2.5	3.75	2.5	EtOH	South	None
10	1	14	5	5	5	EtOH	South	MH
12	1	15	0	0	2.5	Water	South	MH
6	1	16	5	0	5	EtOH	North	LED
24	2	17	0	5	0	EtOH	South	MH
21	2	18	0	0	0	Water	North	MH
15	2	19	0	5	0	Water	South	None
14	2	20	5	2.5	5	EtOH	North	None
13	2	21	5	5	2.5	EtOH	North	None
18	2	22	2.5	0	5	EtOH	South	LED
20	2	23	5	5	0	EtOH	North	MH
17	2	24	0	5	5	EtOH	North	LED
22	2	25	5	0	5	Water	North	MH
24	2	26	0	5	0	EtOH	South	MH
25	2	27	2.5	2.5	5	Water	South	MH
22	2	28	5	0	5	Water	North	MH
16	2	29	0	0	0	EtOH	North	LED
19	2	30	5	0	2.5	Water	South	LED
23	2	31	5	0	0	EtOH	South	MH
27	3	32	5	0	0	Water	North	None
28	3	33	0	0	5	Water	North	None
31	3	34	2.5	1.25	1.25	Water	South	None
32	3	35	0	5	0	Water	North	LED
35	3	36	0	0	0	Water	South	LED
30	3	37	0	5	5	EtOH	South	None
36	3	38	1.25	2.5	1.25	EtOH	North	MH
37	3	39	0	5	5	Water	North	MH
29	3	40	5	5	0	EtOH	South	None
34	3	41	0	2.5	2.5	EtOH	South	LED
26	3	42	2.5	0	2.5	EtOH	North	None
33	3	43	5	5	5	Water	North	LED

*Unique treatment identifier. For example, run #2 and run #8 are replicates and have the same ID of #11.

### Data analysis

Data used for the ANOVA analyses were the means of the 21–28 budwood grafts at each design point. The highest order polynomial where additional terms were significant at the 0.05 level were used for the ANOVA. Data quality and model adequacy tests were conducted and included Box-Cox plots for data transformation requirements, normal probability plots for the residual normality assumption, internally studentized residuals for constant variance assumption, Outlier t-values for outlier identification, lack-of-fit tests for detecting additional signal, predicted vs actual values for unbiased model prediction, Cook’s distance to identify high leverage points, predicted R^2^ to ensure model stability, and adequate precision as a signal to noise measure. The statistical software package Design-Expert 10 (2016) was used to construct the 6-factor RSM design and analyze the data.

### Supplementary Information

Supplementary Table 1.

Supplementary Table 2.

## Data Availability

All data generated or analyzed during this study are included in Supplement Table S1_Data.docx.
