# Supplementary material for: Improving citrus bud grafting efficiency
Source: Sci Rep. 2023 Oct 18;13:17807. doi: 10.1038/s41598-023-44832-x (PMC10584891; doi:10.1038/s41598-023-44832-x)
Supplement: Supplementary file 1 — Supplementary Table 1. [file 41598_2023_44832_MOESM1_ESM.docx]

**Table S1_R1-15.** Data of responses 1-15.

| Design  Points  (Runs) | Responses | | | | | | | | | | | | | | |
| --- | --- | --- | --- | --- | --- | --- | --- | --- | --- | --- | --- | --- | --- | --- | --- |
|  | R1 | R2 | R3 | R4 | R5 | R6 | R7 | R8 | R9 | R10 | R11 | R12 | R13 | R14 | R15 |
| 1 | 2.17 | 1.000 | 0.000 | 0.000 | 0 | 1.000 | 0.000 | 0.000 | 0 | 1.000 | 0.000 | 0.000 | 0 | 1.000 | 0.087 |
| 2 | 1.96 | 1.000 | 0.261 | 0.261 | 38 | 1.000 | 0.261 | 0.261 | 44 | 1.000 | 0.348 | 0.348 | 154 | 1.000 | 0.348 |
| 3 | 1.87 | 1.000 | 0.087 | 0.087 | 19 | 1.000 | 0.261 | 0.261 | 23 | 1.000 | 0.391 | 0.391 | 72 | 1.000 | 0.609 |
| 4 | 2.04 | 1.000 | 0.565 | 0.609 | 46 | 1.000 | 0.696 | 0.652 | 53 | 1.000 | 0.696 | 0.696 | 209 | 1.000 | 0.696 |
| 5 | 2.05 | 1.000 | 0.545 | 0.591 | 35 | 1.000 | 0.591 | 0.591 | 42 | 1.000 | 0.636 | 0.636 | 176 | 1.000 | 0.682 |
| 6 | 2.00 | 0.957 | 0.409 | 0.409 | 43 | 0.957 | 0.455 | 0.455 | 54 | 0.957 | 0.455 | 0.455 | 205 | 0.957 | 0.455 |
| 7 | 1.78 | 1.000 | 0.174 | 0.174 | 47 | 1.000 | 0.174 | 0.174 | 62 | 1.000 | 0.217 | 0.217 | 256 | 1.000 | 0.217 |
| 8 | 2.27 | 1.000 | 0.682 | 0.682 | 39 | 1.000 | 0.727 | 0.727 | 46 | 1.000 | 0.773 | 0.818 | 196 | 1.000 | 0.773 |
| 9 | 1.73 | 1.000 | 0.000 | 0.000 | 0 | 1.000 | 0.000 | 0.000 | 0 | 1.000 | 0.000 | 0.000 | 0 | 1.000 | 0.000 |
| 10 | 2.23 | 1.000 | 0.000 | 0.000 | 0 | 1.000 | 0.273 | 0.273 | 33 | 1.000 | 0.455 | 0.455 | 61 | 1.000 | 0.500 |
| 11 | 2.05 | 1.000 | 0.227 | 0.227 | 25 | 1.000 | 0.455 | 0.455 | 21 | 1.000 | 0.591 | 0.591 | 72 | 1.000 | 0.591 |
| 12 | 1.32 | 1.000 | 0.182 | 0.182 | 27 | 1.000 | 0.182 | 0.182 | 48 | 1.000 | 0.182 | 0.182 | 185 | 1.000 | 0.182 |
| 13 | 1.70 | 1.000 | 0.565 | 0.565 | 13 | 1.000 | 0.522 | 0.522 | 21 | 1.000 | 0.522 | 0.522 | 112 | 1.000 | 0.522 |
| 14 | 2.52 | 1.000 | 0.478 | 0.478 | 30 | 1.000 | 0.522 | 0.565 | 65 | 1.000 | 0.652 | 0.696 | 105 | 1.000 | 0.652 |
| 15 | 2.57 | 1.000 | 0.087 | 0.087 | 4 | 1.000 | 0.174 | 0.174 | 42 | 1.000 | 0.174 | 0.174 | 94 | 1.000 | 0.304 |
| 16 | 2.23 | 1.000 | 0.091 | 0.091 | 4 | 1.000 | 0.136 | 0.136 | 23 | 1.000 | 0.227 | 0.227 | 43 | 1.000 | 0.227 |
| 17 | 1.83 | 1.000 | 0.000 | 0.000 | 0 | 1.000 | 0.042 | 0.042 | 4 | 1.000 | 0.208 | 0.208 | 43 | 1.000 | 0.375 |
| 18 | 2.13 | 1.000 | 0.000 | 0.000 | 0 | 1.000 | 0.000 | 0.000 | 0 | 1.000 | 0.042 | 0.042 | 15 | 1.000 | 0.167 |
| 19 | 1.33 | 1.000 | 0.000 | 0.000 | 0 | 1.000 | 0.000 | 0.000 | 0 | 1.000 | 0.042 | 0.042 | 38 | 1.000 | 0.042 |
| 20 | 1.50 | 1.000 | 0.208 | 0.208 | 30 | 1.000 | 0.208 | 0.208 | 33 | 1.000 | 0.208 | 0.208 | 118 | 1.000 | 0.250 |
| 21 | 1.54 | 1.000 | 0.083 | 0.083 | 8 | 1.000 | 0.125 | 0.125 | 9 | 1.000 | 0.125 | 0.125 | 129 | 1.000 | 0.125 |
| 22 | 2.21 | 1.000 | 0.125 | 0.125 | 12 | 1.000 | 0.208 | 0.208 | 25 | 1.000 | 0.250 | 0.333 | 105 | 1.000 | 0.333 |
| 23 | 2.21 | 1.000 | 0.458 | 0.458 | 44 | 1.000 | 0.458 | 0.458 | 66 | 1.000 | 0.458 | 0.458 | 250 | 1.000 | 0.458 |
| 24 | 2.25 | 1.000 | 0.000 | 0.000 | 0 | 1.000 | 0.000 | 0.000 | 0 | 1.000 | 0.000 | 0.000 | 0 | 1.000 | 0.083 |
| 25 | 2.17 | 1.000 | 0.125 | 0.125 | 56 | 1.000 | 0.125 | 0.125 | 81 | 1.000 | 0.167 | 0.167 | 183 | 1.000 | 0.167 |
| 26 | 2.13 | 1.000 | 0.000 | 0.000 | 0 | 1.000 | 0.083 | 0.083 | 27 | 1.000 | 0.208 | 0.208 | 43 | 1.000 | 0.333 |
| 27 | 2.17 | 0.958 | 0.348 | 0.348 | 46 | 0.958 | 0.348 | 0.348 | 77 | 0.958 | 0.435 | 0.435 | 198 | 0.958 | 0.435 |
| 28 | 2.21 | 1.000 | 0.375 | 0.375 | 45 | 1.000 | 0.375 | 0.375 | 64 | 1.000 | 0.375 | 0.375 | 197 | 1.000 | 0.417 |
| 29 | 1.83 | 1.000 | 0.000 | 0.000 | 0 | 1.000 | 0.000 | 0.000 | 0 | 1.000 | 0.000 | 0.000 | 0 | 1.000 | 0.042 |
| 30 | 1.96 | 1.000 | 0.292 | 0.292 | 53 | 1.000 | 0.292 | 0.292 | 70 | 1.000 | 0.417 | 0.417 | 184 | 1.000 | 0.417 |
| 31 | 1.54 | 1.000 | 0.542 | 0.542 | 42 | 1.000 | 0.542 | 0.542 | 55 | 1.000 | 0.667 | 0.708 | 164 | 1.000 | 0.667 |
| 32 | 1.21 | 1.000 | 0.292 | 0.292 | 40 | 1.000 | 0.292 | 0.292 | 56 | 1.000 | 0.292 | 0.292 | 182 | 1.000 | 0.292 |
| 33 | 1.52 | 1.000 | 0.000 | 0.000 | 0 | 1.000 | 0.043 | 0.043 | 5 | 1.000 | 0.087 | 0.087 | 63 | 1.000 | 0.130 |
| 34  37  38  39  40  41  42  43 | 1.39 | 1.000 | 0.261 | 0.261 | 16 | 1.000 | 0.261 | 0.261 | 21 | 1.000 | 0.261 | 0.261 | 107 | 1.000 | 0.391 |
| 35 | 1.83 | 1.000 | 0.000 | 0.000 | 0 | 1.000 | 0.083 | 0.083 | 13 | 1.000 | 0.083 | 0.083 | 32 | 1.000 | 0.083 |
| 36 | 2.04 | 1.000 | 0.043 | 0.043 | 7 | 1.000 | 0.087 | 0.087 | 8 | 1.000 | 0.217 | 0.217 | 26 | 1.000 | 0.435 |
| 37 | 1.29 | 1.000 | 0.000 | 0.000 | 0 | 1.000 | 0.083 | 0.083 | 20 | 1.000 | 0.208 | 0.208 | 26 | 1.000 | 0.250 |
| 38 | 2.17 | 1.000 | 0.125 | 0.125 | 49 | 1.000 | 0.167 | 0.167 | 47 | 1.000 | 0.167 | 0.167 | 177 | 1.000 | 0.167 |
| 39 | 2.08 | 1.000 | 0.000 | 0.000 | 0 | 1.000 | 0.000 | 0.000 | 0 | 1.000 | 0.083 | 0.083 | 9 | 1.000 | 0.042 |
| 40 | 1.50 | 1.000 | 0.417 | 0.417 | 27 | 1.000 | 0.417 | 0.417 | 35 | 1.000 | 0.458 | 0.542 | 103 | 1.000 | 0.500 |
| 41 | 1.83 | 1.000 | 0.043 | 0.043 | 12 | 1.000 | 0.087 | 0.087 | 9 | 1.000 | 0.304 | 0.348 | 46 | 1.000 | 0.391 |
| 42 | 1.26 | 1.000 | 0.217 | 0.217 | 16 | 1.000 | 0.261 | 0.261 | 27 | 1.000 | 0.261 | 0.261 | 91 | 1.000 | 0.261 |
| 43 | 2.39 | 1.000 | 0.174 | 0.174 | 30 | 1.000 | 0.174 | 0.174 | 47 | 1.000 | 0.174 | 0.174 | 188 | 1.000 | 0.174 |
| Control 1 | 1.65 | 1.000 | 0.000 | 0.000 | 0 | 1.000 | 0.087 | 0.087 | 27 | 1.000 | 0.087 | 0.130 | 29 | 1.000 | 0.261 |
| Control 2 | 1.57 | 1.000 | 0.087 | 0.087 | 11 | 1.000 | 0.087 | 0.087 | 39 | 1.000 | 0.217 | 0.217 | 31 | 1.000 | 0.304 |

Table S1_R1-15 continued

**Table S1_R16-28.** Data of responses 16-28.

| Design  Points  (Runs) | Responses | | | | | | | | | | | | | | |
| --- | --- | --- | --- | --- | --- | --- | --- | --- | --- | --- | --- | --- | --- | --- | --- |
|  | R16 | R17 | R18 | R19 | R20 | R21 | R22 | R23 | R24 | R25 | R26 | R27 | R28 |  |  |
| 1 | 0.087 | 24 | 1.000 | 0.087 | 0.087 | 33 | 22 | 3.5 | 3.5 | 20 | 4.0 | 5.0 | 102 |  |  |
| 2 | 0.391 | 176 | 1.000 | 0.348 | 0.348 | 336 | 241 | 14.5 | 14.3 | 318 | 18.5 | 17.3 | 5317 |  |  |
| 3 | 0.609 | 83 | 1.000 | 0.609 | 0.609 | 154 | 109 | 7.5 | 7.5 | 133 | 8.9 | 13.5 | 3795 |  |  |
| 4 | 0.696 | 243 | 1.000 | 0.696 | 0.696 | 411 | 314 | 18.3 | 18.3 | 314 | 18.3 | 17.1 | 8357 |  |  |
| 5 | 0.727 | 196 | 1.000 | 0.682 | 0.682 | 366 | 283 | 15.1 | 15.1 | 325 | 17.2 | 18.5 | 6535 |  |  |
| 6 | 0.455 | 227 | 0.957 | 0.409 | 0.409 | 421 | 367 | 19.7 | 19.7 | 367 | 19.7 | 18.4 | 8916 |  |  |
| 7 | 0.217 | 260 | 1.000 | 0.217 | 0.217 | 495 | 420 | 21.2 | 21.2 | 420 | 21.2 | 19.6 | 3976 |  |  |
| 8 | 0.773 | 225 | 1.000 | 0.773 | 0.773 | 414 | 352 | 18.1 | 18.1 | 352 | 18.1 | 19.3 | 16548 |  |  |
| 9 | 0.000 | 0 | 1.000 | 0.000 | 0.000 | 0 | 0 | 0.0 | 0.0 | 0 | 0.0 | 0.0 | 0 |  |  |
| 10 | 0.545 | 106 | 1.000 | 0.500 | 0.500 | 220 | 148 | 9.2 | 9.2 | 148 | 9.2 | 16.1 | 2198 |  |  |
| 11 | 0.591 | 103 | 1.000 | 0.591 | 0.591 | 213 | 127 | 8.1 | 8.0 | 182 | 11.0 | 15.1 | 4464 |  |  |
| 12 | 0.182 | 202 | 1.000 | 0.182 | 0.182 | 340 | 265 | 14.5 | 14.3 | 265 | 14.5 | 18.1 | 2434 |  |  |
| 13 | 0.522 | 140 | 1.000 | 0.522 | 0.522 | 288 | 216 | 11.9 | 11.8 | 258 | 14.1 | 17.0 | 4794 |  |  |
| 14 | 0.652 | 169 | 1.000 | 0.652 | 0.652 | 286 | 223 | 13.7 | 13.7 | 238 | 14.5 | 15.4 | 7605 |  |  |
| 15 | 0.304 | 119 | 1.000 | 0.304 | 0.304 | 206 | 146 | 10.3 | 10.3 | 170 | 11.7 | 11.9 | 2484 |  |  |
| 16 | 0.227 | 124 | 1.000 | 0.227 | 0.227 | 202 | 182 | 11.6 | 11.6 | 227 | 14.3 | 15.7 | 250 |  |  |
| 17 | 0.375 | 61 | 1.000 | 0.667 | 0.667 | 96 | 47 | 3.6 | 3.6 | 91 | 6.9 | 12.1 | 1710 |  |  |
| 18 | 0.167 | 16 | 1.000 | 0.125 | 0.125 | 104 | 59 | 4.3 | 4.3 | 84 | 6.0 | 11.8 | 282 |  |  |
| 19 | 0.042 | 63 | 1.000 | 0.167 | 0.208 | 66 | 15 | 1.3 | 1.3 | 58 | 5.0 | 11.6 | 164 |  |  |
| 20 | 0.250 | 142 | 1.000 | 0.250 | 0.250 | 263 | 157 | 9.8 | 9.8 | 188 | 11.6 | 15.8 | 1494 |  |  |
| 21 | 0.125 | 182 | 1.000 | 0.125 | 0.125 | 274 | 226 | 14.0 | 14.0 | 226 | 14.0 | 15.9 | 1049 |  |  |
| 22 | 0.333 | 136 | 1.000 | 0.333 | 0.333 | 236 | 185 | 11.4 | 11.4 | 208 | 12.6 | 15.9 | 2681 |  |  |
| 23 | 0.458 | 289 | 1.000 | 0.458 | 0.458 | 457 | 368 | 21.1 | 21.1 | 368 | 21.1 | 17.0 | 8854 |  |  |
| 24 | 0.083 | 31 | 1.000 | 0.125 | 0.125 | 29 | 26 | 3.0 | 3.0 | 39 | 4.5 | 8.5 | 212 |  |  |
| 25 | 0.167 | 210 | 1.000 | 0.167 | 0.167 | 413 | 332 | 18.0 | 18.0 | 441 | 23.7 | 18.6 | 2569 |  |  |
| 26 | 0.375 | 51 | 1.000 | 0.500 | 0.500 | 110 | 62 | 4.0 | 4.0 | 144 | 8.6 | 17.0 | 1581 |  |  |
| 27 | 0.435 | 248 | 0.958 | 0.478 | 0.478 | 367 | 290 | 17.1 | 17.1 | 354 | 20.7 | 17.2 | 7582 |  |  |
| 28 | 0.417 | 248 | 1.000 | 0.417 | 0.417 | 364 | 344 | 20.4 | 20.4 | 382 | 22.6 | 16.5 | 8431 |  |  |
| 29 | 0.042 | 41 | 1.000 | 0.042 | 0.042 | 86 | 38 | 4.0 | 4.0 | 38 | 4.0 | 9.5 | 47 |  |  |
| 30 | 0.417 | 209 | 1.000 | 0.458 | 0.458 | 322 | 258 | 14.6 | 14.6 | 284 | 16.1 | 17.3 | 5700 |  |  |
| 31 | 0.667 | 189 | 1.000 | 0.750 | 0.750 | 298 | 252 | 14.3 | 14.3 | 323 | 18.2 | 17.5 | 8906 |  |  |
| 32 | 0.333 | 219 | 1.000 | 0.292 | 0.292 | 355 | 295 | 17.7 | 17.7 | 295 | 17.7 | 16.2 | 4120 |  |  |
| 33 | 0.130 | 59 | 1.000 | 0.174 | 0.174 | 125 | 56 | 4.3 | 4.0 | 109 | 7.0 | 14.7 | 275 |  |  |
| 34  37  38  39  40  41  42  43 | 0.391 | 98 | 1.000 | 0.348 | 0.348 | 177 | 109 | 8.0 | 8.0 | 144 | 10.2 | 13.3 | 2100 |  |  |
| 35 | 0.083 | 140 | 1.000 | 0.125 | 0.125 | 149 | 94 | 7.0 | 7.0 | 142 | 10.5 | 13.5 | 573 |  |  |
| 36 | 0.435 | 37 | 1.000 | 0.565 | 0.565 | 107 | 44 | 3.6 | 3.6 | 100 | 7.0 | 14.0 | 1207 |  |  |
| 37 | 0.250 | 60 | 1.000 | 0.208 | 0.208 | 144 | 70 | 5.4 | 5.4 | 161 | 10.5 | 15.7 | 689 |  |  |
| 38 | 0.167 | 187 | 1.000 | 0.167 | 0.167 | 321 | 237 | 13.5 | 13.5 | 312 | 17.3 | 18.0 | 1526 |  |  |
| 39 | 0.042 | 23 | 1.000 | 0.208 | 0.208 | 48 | 4 | 0.4 | 0.4 | 0 | 0.0 | 0.0 | 115 |  |  |
| 40 | 0.500 | 145 | 1.000 | 0.500 | 0.500 | 221 | 166 | 10.8 | 10.8 | 181 | 11.5 | 15.5 | 3203 |  |  |
| 41 | 0.391 | 52 | 1.000 | 0.435 | 0.435 | 141 | 59 | 4.5 | 4.5 | 109 | 7.6 | 13.1 | 1047 |  |  |
| 42 | 0.261 | 154 | 1.000 | 0.261 | 0.261 | 246 | 196 | 12.5 | 12.5 | 196 | 12.5 | 15.2 | 1773 |  |  |
| 43 | 0.174 | 257 | 1.000 | 0.174 | 0.174 | 412 | 339 | 19.8 | 19.8 | 339 | 19.8 | 17.0 | 2903 |  |  |
| Control 1 | 0.261 | 34 | 1.000 | 0.304 | 0.304 | 70 | 28 | 3.4 | 3.4 | 50 | 5.0 | 9.3 | 671 |  |  |
| Control 2 | 0.304 | 75 | 1.000 | 0.478 | 0.478 | 85 | 54 | 4.3 | 4.3 | 113 | 8.8 | 12.7 | 1058 |  |  |

Table S1_R16-28 continued
