# Supplementary material for: Improving citrus bud grafting efficiency
Source: Sci Rep. 2023 Oct 18;13:17807. doi: 10.1038/s41598-023-44832-x (PMC10584891; doi:10.1038/s41598-023-44832-x)
Supplement: Supplementary file 2 — Supplementary Table 2. [file 41598_2023_44832_MOESM2_ESM.docx]

**Table S2.** Pearson correlation coefficient (r) responses 1 – 28.

|  | 1 | 2 | 3 | 4 | 5 | 6 | 7 | 8 | 9 | 10 | 11 | 12 | 13 | 14 | 15 |
| --- | --- | --- | --- | --- | --- | --- | --- | --- | --- | --- | --- | --- | --- | --- | --- |
| 1 | 1 | -0.11 | 0.07 | 0.07 | 0.11 | -0.11 | 0.13 | 0.14 | 0.23 | -0.11 | 0.17 | 0.16 | 0.07 | -0.11 | 0.2 |
| 2 | -0.11 | 1 | -0.2 | -0.2 | -0.27 | 1 | -0.18 | -0.18 | -0.31 | 1 | -0.16 | -0.15 | -0.27 | 1 | -0.12 |
| 3 | 0.07 | -0.2 | 1 | 1 | 0.7 | -0.2 | 0.95 | 0.95 | 0.64 | -0.2 | 0.87 | 0.86 | 0.71 | -0.2 | 0.77 |
| 4 | 0.07 | -0.2 | 1 | 1 | 0.7 | -0.2 | 0.95 | 0.95 | 0.63 | -0.2 | 0.87 | 0.86 | 0.7 | -0.2 | 0.77 |
| 5 | 0.11 | -0.27 | 0.7 | 0.7 | 1 | -0.27 | 0.64 | 0.64 | 0.9 | -0.27 | 0.58 | 0.57 | 0.91 | -0.27 | 0.46 |
| 6 | -0.11 | 1 | -0.2 | -0.2 | -0.27 | 1 | -0.18 | -0.18 | -0.31 | 1 | -0.16 | -0.15 | -0.27 | 1 | -0.12 |
| 7 | 0.13 | -0.18 | 0.95 | 0.95 | 0.64 | -0.18 | 1 | 1 | 0.61 | -0.18 | 0.96 | 0.95 | 0.65 | -0.18 | 0.88 |
| 8 | 0.14 | -0.18 | 0.95 | 0.95 | 0.64 | -0.18 | 1 | 1 | 0.62 | -0.18 | 0.96 | 0.95 | 0.65 | -0.18 | 0.88 |
| 9 | 0.23 | -0.31 | 0.64 | 0.63 | 0.9 | -0.31 | 0.61 | 0.62 | 1 | -0.31 | 0.57 | 0.56 | 0.88 | -0.31 | 0.46 |
| 10 | -0.11 | 1 | -0.2 | -0.2 | -0.27 | 1 | -0.18 | -0.18 | -0.31 | 1 | -0.16 | -0.15 | -0.27 | 1 | -0.12 |
| 11 | 0.17 | -0.16 | 0.87 | 0.87 | 0.58 | -0.16 | 0.96 | 0.96 | 0.57 | -0.16 | 1 | 1 | 0.55 | -0.16 | 0.96 |
| 12 | 0.16 | -0.15 | 0.86 | 0.86 | 0.57 | -0.15 | 0.95 | 0.95 | 0.56 | -0.15 | 1 | 1 | 0.53 | -0.15 | 0.95 |
| 13 | 0.07 | -0.27 | 0.71 | 0.7 | 0.91 | -0.27 | 0.65 | 0.65 | 0.88 | -0.27 | 0.55 | 0.53 | 1 | -0.27 | 0.43 |
| 14 | -0.11 | 1 | -0.2 | -0.2 | -0.27 | 1 | -0.18 | -0.18 | -0.31 | 1 | -0.16 | -0.15 | -0.27 | 1 | -0.12 |
| 15 | 0.2 | -0.12 | 0.77 | 0.77 | 0.46 | -0.12 | 0.88 | 0.88 | 0.46 | -0.12 | 0.96 | 0.95 | 0.43 | -0.12 | 1 |
| 16 | 0.2 | -0.12 | 0.76 | 0.76 | 0.46 | -0.12 | 0.87 | 0.88 | 0.47 | -0.12 | 0.95 | 0.95 | 0.43 | -0.12 | 1 |
| 17 | 0.11 | -0.27 | 0.7 | 0.7 | 0.86 | -0.27 | 0.66 | 0.66 | 0.88 | -0.27 | 0.55 | 0.54 | 0.96 | -0.27 | 0.41 |
| 18 | -0.11 | 1 | -0.2 | -0.2 | -0.27 | 1 | -0.18 | -0.18 | -0.31 | 1 | -0.16 | -0.15 | -0.27 | 1 | -0.12 |
| 19 | 0.19 | -0.09 | 0.66 | 0.67 | 0.36 | -0.09 | 0.77 | 0.77 | 0.37 | -0.09 | 0.88 | 0.88 | 0.33 | -0.09 | 0.95 |
| 20 | 0.19 | -0.09 | 0.66 | 0.66 | 0.36 | -0.09 | 0.77 | 0.77 | 0.36 | -0.09 | 0.88 | 0.88 | 0.32 | -0.09 | 0.95 |
| 21 | 0.13 | -0.26 | 0.71 | 0.71 | 0.88 | -0.26 | 0.68 | 0.68 | 0.88 | -0.26 | 0.59 | 0.58 | 0.97 | -0.26 | 0.46 |
| 22 | 0.16 | -0.28 | 0.71 | 0.71 | 0.87 | -0.28 | 0.67 | 0.67 | 0.88 | -0.28 | 0.57 | 0.55 | 0.96 | -0.28 | 0.44 |
| 23 | 0.17 | -0.27 | 0.7 | 0.7 | 0.85 | -0.27 | 0.67 | 0.67 | 0.88 | -0.27 | 0.56 | 0.55 | 0.95 | -0.27 | 0.43 |
| 24 | 0.17 | -0.27 | 0.7 | 0.7 | 0.85 | -0.27 | 0.67 | 0.67 | 0.88 | -0.27 | 0.56 | 0.55 | 0.95 | -0.27 | 0.44 |
| 25 | 0.16 | -0.28 | 0.68 | 0.68 | 0.88 | -0.28 | 0.64 | 0.64 | 0.9 | -0.28 | 0.57 | 0.55 | 0.94 | -0.28 | 0.45 |
| 26 | 0.16 | -0.28 | 0.67 | 0.66 | 0.87 | -0.28 | 0.63 | 0.63 | 0.9 | -0.28 | 0.56 | 0.54 | 0.92 | -0.28 | 0.44 |
| 27 | -0.01 | -0.16 | 0.54 | 0.53 | 0.64 | -0.16 | 0.57 | 0.57 | 0.68 | -0.16 | 0.57 | 0.56 | 0.73 | -0.16 | 0.53 |
| 28 | 0.26 | -0.31 | 0.88 | 0.87 | 0.7 | -0.31 | 0.88 | 0.88 | 0.68 | -0.31 | 0.85 | 0.84 | 0.71 | -0.31 | 0.78 |

Table S2 continued

|  | 16 | 17 | 18 | 19 | 20 | 21 | 22 | 23 | 24 | 25 | 26 | 27 | 28 |  |  |
| --- | --- | --- | --- | --- | --- | --- | --- | --- | --- | --- | --- | --- | --- | --- | --- |
| 1 | 0.2 | 0.11 | -0.11 | 0.19 | 0.19 | 0.13 | 0.16 | 0.17 | 0.17 | 0.16 | 0.16 | -0.01 | 0.26 |  |  |
| 2 | -0.12 | -0.27 | 1 | -0.09 | -0.09 | -0.26 | -0.28 | -0.27 | -0.27 | -0.28 | -0.28 | -0.16 | -0.31 |  |  |
| 3 | 0.76 | 0.7 | -0.2 | 0.66 | 0.66 | 0.71 | 0.71 | 0.7 | 0.7 | 0.68 | 0.67 | 0.54 | 0.88 |  |  |
| 4 | 0.76 | 0.7 | -0.2 | 0.67 | 0.66 | 0.71 | 0.71 | 0.7 | 0.7 | 0.68 | 0.66 | 0.53 | 0.87 |  |  |
| 5 | 0.46 | 0.86 | -0.27 | 0.36 | 0.36 | 0.88 | 0.87 | 0.85 | 0.85 | 0.88 | 0.87 | 0.64 | 0.7 |  |  |
| 6 | -0.12 | -0.27 | 1 | -0.09 | -0.09 | -0.26 | -0.28 | -0.27 | -0.27 | -0.28 | -0.28 | -0.16 | -0.31 |  |  |
| 7 | 0.87 | 0.66 | -0.18 | 0.77 | 0.77 | 0.68 | 0.67 | 0.67 | 0.67 | 0.64 | 0.63 | 0.57 | 0.88 |  |  |
| 8 | 0.88 | 0.66 | -0.18 | 0.77 | 0.77 | 0.68 | 0.67 | 0.67 | 0.67 | 0.64 | 0.63 | 0.57 | 0.88 |  |  |
| 9 | 0.47 | 0.88 | -0.31 | 0.37 | 0.36 | 0.88 | 0.88 | 0.88 | 0.88 | 0.9 | 0.9 | 0.68 | 0.68 |  |  |
| 10 | -0.12 | -0.27 | 1 | -0.09 | -0.09 | -0.26 | -0.28 | -0.27 | -0.27 | -0.28 | -0.28 | -0.16 | -0.31 |  |  |
| 11 | 0.95 | 0.55 | -0.16 | 0.88 | 0.88 | 0.59 | 0.57 | 0.56 | 0.56 | 0.57 | 0.56 | 0.57 | 0.85 |  |  |
| 12 | 0.95 | 0.54 | -0.15 | 0.88 | 0.88 | 0.58 | 0.55 | 0.55 | 0.55 | 0.55 | 0.54 | 0.56 | 0.84 |  |  |
| 13 | 0.43 | 0.96 | -0.27 | 0.33 | 0.32 | 0.97 | 0.96 | 0.95 | 0.95 | 0.94 | 0.92 | 0.73 | 0.71 |  |  |
| 14 | -0.12 | -0.27 | 1 | -0.09 | -0.09 | -0.26 | -0.28 | -0.27 | -0.27 | -0.28 | -0.28 | -0.16 | -0.31 |  |  |
| 15 | 1 | 0.41 | -0.12 | 0.95 | 0.95 | 0.46 | 0.44 | 0.43 | 0.44 | 0.45 | 0.44 | 0.53 | 0.78 |  |  |
| 16 | 1 | 0.4 | -0.12 | 0.95 | 0.95 | 0.46 | 0.43 | 0.43 | 0.43 | 0.45 | 0.44 | 0.53 | 0.77 |  |  |
| 17 | 0.4 | 1 | -0.27 | 0.3 | 0.29 | 0.97 | 0.97 | 0.98 | 0.98 | 0.94 | 0.95 | 0.74 | 0.7 |  |  |
| 18 | -0.12 | -0.27 | 1 | -0.09 | -0.09 | -0.26 | -0.28 | -0.27 | -0.27 | -0.28 | -0.28 | -0.16 | -0.31 |  |  |
| 19 | 0.95 | 0.3 | -0.09 | 1 | 1 | 0.33 | 0.3 | 0.29 | 0.3 | 0.33 | 0.32 | 0.44 | 0.7 |  |  |
| 20 | 0.95 | 0.29 | -0.09 | 1 | 1 | 0.33 | 0.3 | 0.29 | 0.29 | 0.33 | 0.32 | 0.44 | 0.7 |  |  |
| 21 | 0.46 | 0.97 | -0.26 | 0.33 | 0.33 | 1 | 0.99 | 0.98 | 0.98 | 0.97 | 0.96 | 0.79 | 0.71 |  |  |
| 22 | 0.43 | 0.97 | -0.28 | 0.3 | 0.3 | 0.99 | 1 | 0.99 | 0.99 | 0.97 | 0.96 | 0.75 | 0.72 |  |  |
| 23 | 0.43 | 0.98 | -0.27 | 0.29 | 0.29 | 0.98 | 0.99 | 1 | 1 | 0.96 | 0.96 | 0.75 | 0.71 |  |  |
| 24 | 0.43 | 0.98 | -0.27 | 0.3 | 0.29 | 0.98 | 0.99 | 1 | 1 | 0.96 | 0.96 | 0.75 | 0.71 |  |  |
| 25 | 0.45 | 0.94 | -0.28 | 0.33 | 0.33 | 0.97 | 0.97 | 0.96 | 0.96 | 1 | 0.99 | 0.82 | 0.7 |  |  |
| 26 | 0.44 | 0.95 | -0.28 | 0.32 | 0.32 | 0.96 | 0.96 | 0.96 | 0.96 | 0.99 | 1 | 0.82 | 0.68 |  |  |
| 27 | 0.53 | 0.74 | -0.16 | 0.44 | 0.44 | 0.79 | 0.75 | 0.75 | 0.75 | 0.82 | 0.82 | 1 | 0.53 |  |  |
| 28 | 0.77 | 0.7 | -0.31 | 0.7 | 0.7 | 0.71 | 0.72 | 0.71 | 0.71 | 0.7 | 0.68 | 0.53 | 1 |  |  |
